# Near-threshold fatigue crack propagation without oxide-induced crack closure

**DOI:** 10.1038/s41598-020-64915-3

**Published:** 2020-05-13

**Authors:** Koki Tazoe, Hiroto Tanaka, Masanori Oka, Genki Yagawa

**Affiliations:** 1 Research and Development Center, YANMAR HOLDINGS CO., LTD., 2481, Umegahara, Maibara, Shiga, 521-8511 Japan; 2 University of Tokyo and Toyo University, c/o Toyo University, Kawagoe, Saitama, 350-8585 Japan

**Keywords:** Engineering, Mechanical engineering

## Abstract

An accurate value for the threshold stress intensity factor range, Δ*K*_th_, is a key parameter for studying crack-like defects. However, it is difficult to obtain accurate Δ*K*_th_ values due to oxide-induced crack closure. In this study, we report conditions for minimizing the effects of oxide-induced crack closure near the threshold region, where a concave curve of the effect on the loading frequency on oxide-induced crack closure is achieved. The resulting conditions allow for an accurate determination of Δ*K*_th_, which is a key material parameter relating to the pertinent loading ratio.

## Introduction

Almost all the mechanical structures are exposed to cyclic loading, which means that it is important to estimate the fatigue strength of mechanical structures to ensure safe operation. It is also well known that obtaining an accurate value for the threshold stress intensity factor range, Δ*K*_th_, is necessary for studying fatigue related problems.

Δ*K*_th_ is typically obtained via the Δ*K*-decreasing test based on ASTM E647 standard^1^, that the force is controlled to decrease step-by-step as the crack grows in the fatigue test, which is known to be affected by plasticity-induced^2–7^, roughness-induced^2,8–12^ and oxide-induced crack closures^2,4,5,7,11,13–31^. Among these types of crack closures, the roughness-induced closure is thought to be a material property because the roughness of the fracture surface is related to the microstructure of the material^10^. In contrast, the plasticity-induced and the oxide-induced closures are considered to be dependent on the testing conditions^2–5,11,13–28^. In order to obtain an accurate Δ*K*_th_ value, these types of closures should be minimized.

Many studies have been performed on the plasticity-induced closure^32–35^ and have been summarized in the ASTM standard^1^. The oxide-induced closure has also been studied in order to discuss the effects of various parameters^2,4,5,11,13–31^. However, to the best of our knowledge, there have been no studies on the conditions that are necessary to minimize the oxide-induced closure at a low stress ratio when tested in air.

According previous studies, it is considered that the large amount of oxides is generated on fracture surfaces by fretting oxidation^18^, causing the closure effect due to pushing up the crack closing point^2^. Regarding the causes for the oxidation, the humidity^19,29^ and the loading frequency^15,22,27,30^ are important factors.

Since high-moisture conditions accelerate oxidation, a low-humidity condition is recommended to minimize the oxide-induced closure. The effects of the loading frequency on the oxide-induced closure were studied by Bignonnet *et al*.^22^, who found that the magnitude of the closure in a structural steel at 7 Hz is smaller than that at 65 Hz. Todd *et al*^30^. also reported a similar trend based on results from a MIL-S-24645 base metal. Conversely, Radon^36^ reported that the Δ*K*_th_ of an aluminium alloy tested at a high frequency tends to be smaller than that at a low frequency based on test results at 35 and 0.15 Hz. Skelton and Haigh^15^ also reported the same tendency based on results from a Cr-Mo-V steel at a high temperature and 10 to 0.01 Hz conditions. These studies, although they are contradictory, clearly show that the loading frequency significantly affects the oxide-induced closure.

On the other hand, Tazoe *et al*^37^. reported that no oxides can be clearly observed on the near-threshold fracture surface of a low alloy steel tested at 5 Hz in air. In contrast, Suresh *et al*^20^. reported that for a similar material tested at 50 Hz that the oxides can clearly be observed on the fracture surface. In addition, Tkach and Lenets^27^ tested a similar material with Δ*K* = 9 MPa m^1/2^ reporting that the clear oxides are observed on the fracture surfaces when tested at 15 Hz and 7.5 Hz but not at 0.15 Hz. Accordingly, we could create a hypothesis that the oxidation is minimal near 5 Hz.

To investigate the validity of the hypothesis and to study if the same hypothesis is applicable to other iron-based structural materials, the Δ*K*-decreasing tests^1^ for three different iron-based materials were carried out.

## Results

### Observation of macroscopic fracture surfaces

**Figure 1:**
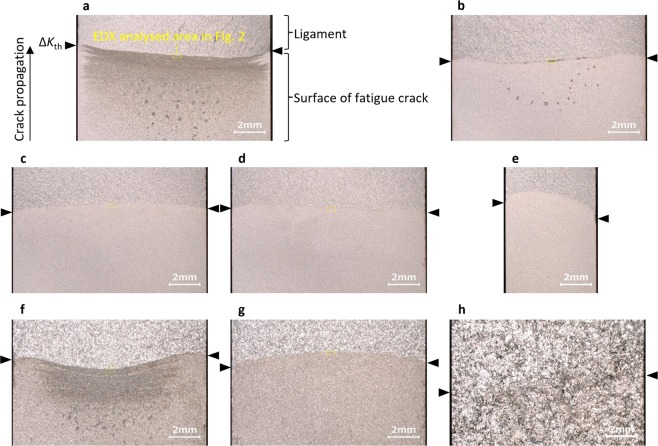
Macroscopic fracture surfaces. Low alloy steel tested at (**a)** 20 Hz, (**b)** 10 Hz, (**c**) 5 Hz, (**d)** 3 Hz and (**e)** 5 Hz. Carbon steel tested at (**f**) 10 Hz and (**g**) 5 Hz. Cast iron tested at (**h**) 20 Hz.

Figure 1 shows the macroscopic fracture surfaces of a low alloy steel (JIS-SCM440), a carbon steel (JIS-S50C) and cast iron. As seen in Fig. 1, the cracks propagate from the bottom to the top and the final crack-front lines, where Δ*K* = Δ*K*_th_, are indicated by triangles.

In the case of the low alloy steel, at 20 Hz (see Fig. 1a), the oxides, which are the debris with diameters of 0.1 mm, are distributed near the centre of the fracture surface. Additionally, the belt-like oxides can be observed across the whole fracture surface. At 10 Hz (see Fig. 1b), the oxide debris can still be seen, but a smaller amount is present than was seen at 20 Hz. In contrast, at 5 and 3 Hz (see Fig. 1c–e, respectively), no oxides are observed, and this result does not depend on the specimen thickness. Therefore, these results clearly show that decreasing the loading frequency decreases the amount of oxides on the fracture surface, and the clearly observable oxides disappear at approximately 5 Hz.

In the case of the carbon steel, at 10 Hz (see Fig. 1f) the oxides can clearly be seen at the centre of the fracture surface in larger quantities than was observed in the low alloy steel. At 5 Hz (see Fig. 1g) no oxides are observed, which is similar to the low alloy steel.

In case of the cast iron, it is difficult to find any oxides or crack-front shapes on the macroscopic fracture surface (see Fig. 1h). In the next subsection, the crack-front lines were analysed in detail by scanning electron microscope (SEM) before energy-dispersive X-ray spectrometry (EDX) analysis was performed.

### EDX analysis

**Figure 2:**
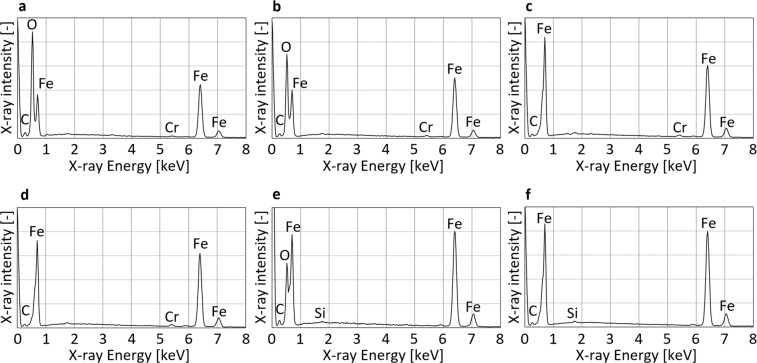
EDX analysis results of the low alloy steel and the carbon steel samples. (**a**) crack-front area of Fig. 1a, (**b**) the area of Fig. 1b, (**c**) the area of Fig. 1c, (**d**) the area of Fig. 1d, (**e**) the area of Fig. 1f and (**f**) the area of Fig. 1g.

Figure 2 shows the EDX analysis results of the fracture surfaces of the low alloy steel and the carbon steel shown above. The analysed areas were approximately 0.6 mm in the direction of the specimen thickness and 0.3 mm in the direction of the crack propagation near the crack-front region (see Fig. 1). For each fracture surface, a few areas near the centre region were analysed and the representative histograms are shown in the figure.

For the low alloy steel, a clear oxygen peak is present for both the 20 and 10 Hz samples (see Fig. 2a and b, respectively), and the oxides can clearly be seen. In contrast, no oxygen peak was detected for the 5 or 3 Hz samples (see Fig. 2c and d, respectively).

Similarly, for the carbon steel, a clear oxygen peak was detected for the 10 Hz sample, and the oxides are visible, but no oxygen peak was detected for the 5 Hz sample (see Fig. 2e and f for the data at 10 Hz and 5 Hz, respectively).

**Figure 3:**
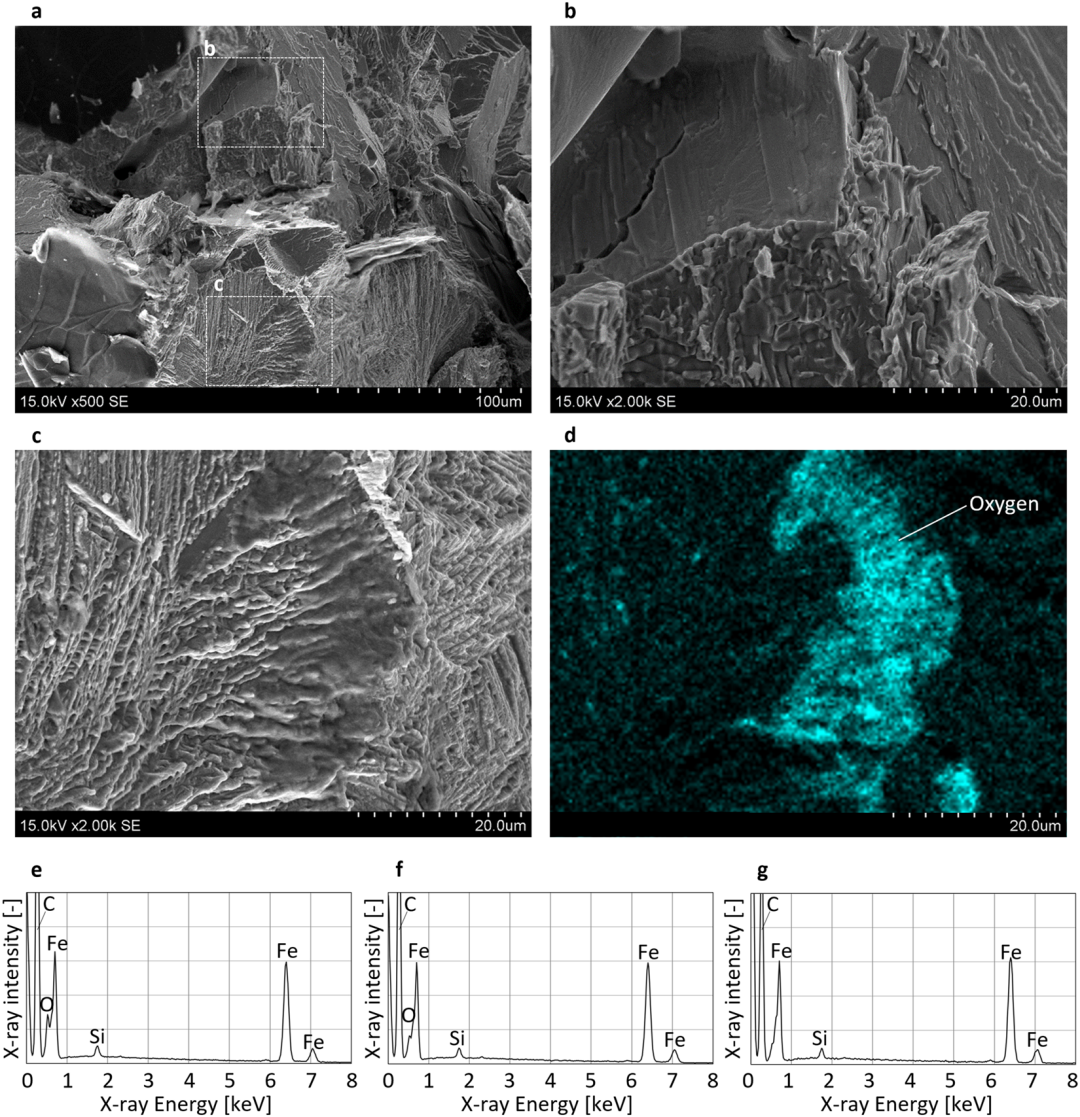
SEM images and EDX analysis results of the cast iron sample. (**a**) crack-front area of Fig. 1h. (**b** and **c**) detailed images of **a**. (**d**) distribution of oxygen in **c**. (**e**) EDX analysis result of crack-front area tested at 20 Hz (shown in Fig. 1h), (**f**) 10 Hz and (**g**) 5 Hz.

Figure 3 shows SEM images and the EDX analysis results for the cast iron. Figure 3a–c show the fracture surfaces near the crack-front area in the centre region of Fig. 1h. Crack-front lines are visible at the border of the brittle region (see Fig. 3b) and the fatigue (see Fig. 3c) fracture surfaces. Furthermore, for the 20 Hz sample the clear oxides are present on the near threshold region (see Fig. 3c and d, respectively).

A clear oxygen peak is present for the 20 Hz cast iron sample (see Fig. 3e), and a small peak is also observed for the 10 Hz sample (see Fig. 3f). Similar to the results from the other materials, no oxygen peak was detected for the 5 Hz sample (see Fig. 3g).

As shown in the above figures, no oxides were detected by EDX for the fracture surfaces of any of the samples tested at 5 and 3 Hz. It is believed that new fracture surfaces created during fatigue tests in air should be oxidized immediately, however, as the macroscopic fracture surfaces show, natural oxides are only observed in very low quantity. Thus, they were not detectable by EDX.

### Fatigue crack propagation

According to the above results, excess oxides were not present on the fracture surfaces when the samples were tested at approximately 5 Hz, and the same trend was observed in all the tested materials. Therefore, in this subsection the threshold phenomenon without the presence of excess oxides is discussed.

**Figure 4:**
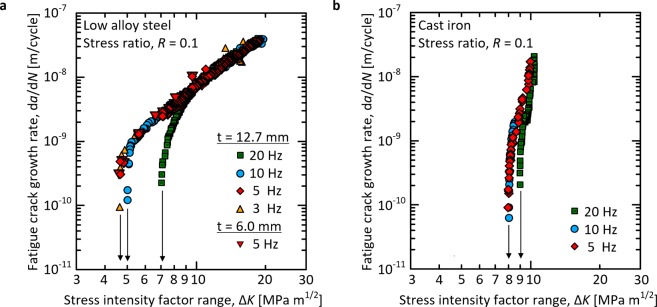
Relationships between d*a*/d*N* and Δ*K*. (**a**) low alloy steel and (**b**) cast iron.

Figure 4a shows the relationship between the fatigue crack growth rate, d*a*/d*N*, and Δ*K* for the low alloy steel. For the 20 and 10 Hz samples, i.e., the conditions that clearly produce the oxides, d*a*/d*N* decreases significantly at 9.0 and 5.4 MPa m^1/2^, and the threshold behaviour appears at 7.1 and 5.0 MPa m^1/2^, respectively. On the other hand, for the 5 Hz and 3 Hz samples, i.e., the conditions without the oxides, d*a*/d*N* is almost the same across the whole Δ*K* region, and the threshold behaviour appears at almost the same Δ*K* value (4.6 MPa m^1/2^).

Figure 4a also shows that two samples with different thicknesses (12.7 and 6 mm) have almost the same d*a*/d*N* curves and the same threshold behaviour. Past studies have reported that the threshold behaviour is affected by the specimen thickness due to differences in the oxide-induced closure^26,31^. However, in the present conditions without the presence of excess oxides, the difference in behaviour for the 12.7 and 6 mm low alloy steels clearly disappears.

Figure 4b shows the results for the cast iron samples. At 20 Hz, the oxides are clearly visible and the threshold behaviour appears at 9.0 MPa m^1/2^. For the 10 Hz and 5 Hz samples where the oxides are only present in small amounts or not at all, the d*a*/d*N* values show nearly the same results, and the same threshold behaviours appear at Δ*K* = 8.0 MPa m^1/2^.

Based on these results, the use of a loading frequency of approximately 5 Hz for the above iron-based materials does not induce the oxide-induced closure and produces thresholds equivalent to the material properties under the same loading ratio. The above results also suggest the possibility that other iron-based materials have similar properties.

## Discussion

The above results show that the amount of oxides on the fracture surfaces of iron-based materials can be reduced by decreasing the loading frequency, and the oxides disappear completely at approximately 5 Hz. Based on the above findings, it is necessary to discuss the mechanism behind the disappearance of the oxides and the effect of the loading frequency has on this mechanism in relation to the results of past studies.

According to previous studies, the main cause of the oxide-induced closure is fretting oxidation due to fracture surfaces smashing into each other^18^. Therefore, it is reasonable to think that the mechanism behind the disappearance of the oxides is related to the mechanism of fretting oxidation.

**Figure 5:**
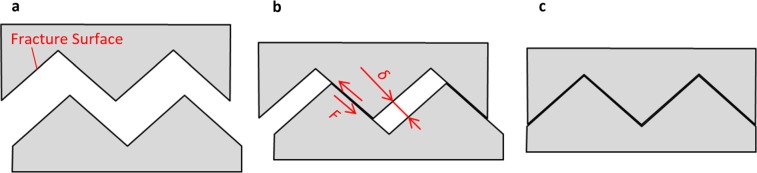
Schematics of smashing fracture surfaces. Schematics of (**a**) crack opening, (**b**) contacting and (**c**) closing.

Schematics that show fracture surfaces smashing into one another are shown in Fig. 5. During the process of unloading in a fatigue cycle, the gap between the fracture surfaces decreases and the fracture surfaces make contact due to the roughness-induced closure^2,8–12^ and the fracture surfaces start grinding against each other. Minakawa and McEvily^9^ emphasized that this smashing model contains mode II loading. However, according to *in situ* observations using atomic force microscopy made by Oda *et al*.^38,39^, Sugeta *et al*^40^. and Jono *et al*.^41^, the fracture surfaces are offset in the direction of the crack propagation during glide plane decohesion^42^ under mode I loading. In addition, Tomlinson *et al*^43^. reported that fretting could occur due to a short slip distance, such as a few nanometres. Therefore, it is thought that the models shown in Fig. 5 are also valid for mode I loading. Next, the mechanism of the disappearance of the oxides is discussed in relation to the smashing model.

The quantity of heat generated during a fatigue cycle due to the friction between the fracture surfaces, *Q*_cy_, is calculated as follows:

1 $${Q}_{cy}\approx F\delta $$

where *F* and *δ* are the friction force and the distance of the slip on the surfaces, respectively. Here, *F* is calculated as follows:

2 $$F\approx \mu P$$

where *μ* and *P* are the coefficient of friction and the vertical force on the fracture surfaces, respectively. For the above equations, the characteristics of the fracture surfaces are related to the microstructures of the materials;^10^ in other words, *μ* and *δ* are considered material properties. In addition, *P* is related to the amount of plasticity-induced closure^2–7^, which means *P* is related to the yield stress and the Young’s modulus. Therefore, under the same loading conditions, *P* is also considered a material property. Accordingly, *Q*_cy_ must then also be a material property, and the quantity of heat released in a unit of time, *Q*_ut_, should be proportional to the loading frequency *f* as follows:

3 $${Q}_{ut}\approx {Q}_{cy}f$$

Consequently, reducing *f*, which is a reduction of *Q*_ut_, is the main cause behind the disappearance of the oxides. Furthermore, it is assumed that the difference in the magnitude of oxidation of the low alloy steel and the carbon steel is caused by differences in the materials’ properties, such as *δ* and *P*.

If the above hypotheses are correct, an increase in the other fretting parameters would create the oxides at the 5 Hz condition. To verify the above hypotheses, an additional Δ*K*-decreasing test was carried out with the low alloy steel at the 5 Hz condition. In this additional test, a small amount of mode III loading was added by twisting the positions of the loading pins. Due to this twisting, *δ* was specifically increased.

**Figure 6:**
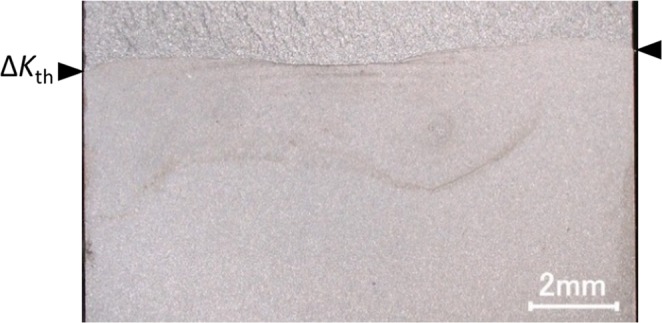
The macroscopic fracture surface of the additional test sample.

Figure 6 shows the macroscopic fracture surface of this tested specimen. As shown in the figure, the belt-like oxides are clearly visible at the centre of the fracture surface. Moreover, the crack-front shape is slightly concave, which indicates that the oxides are likely inducing closure in that location. Therefore, increasing the above fretting parameters creates oxides, and the amount of oxides produced is strongly related to *Q*_ut_.

Furthermore, it is thought that a decrease in *Q*_ut_ indicates a decrease in the temperature of the fracture surface. Benoit *et al*^18^. observed a crack tip in a stainless steel by thermography and reported that a large amount of thermal flux, which is equivalent to 423 K or more, was not detected. Loos and Brotzen^44^ reported the same result. However, as suggested by Tkach and Lenets^27^, temperature increases due to the smashing of the fracture surfaces might occur locally. Moreover, as shown in Fig. 1a,b, f and  3c, d, the oxides, at the centre of the specimen thickness also suggest that local heating might occur there. However, it is difficult to observe such local temperatures in detail, especially in the centre of the specimen thickness, with contemporary techniques. We hope that technological advances will enable us to elucidate these phenomena in the future.

In contrast to the results of this study, some past reports have shown that the magnitude of oxide-induced closure increases by decreasing the loading frequency^15,36^. For example, Radon^36^ tested an aluminium alloy at several loading frequency conditions (35 and 0.15 Hz) and reported that the Δ*K*_th_ at low frequencies is higher than that at high frequencies. In addition, Skelton and Haigh^15^ reported the same result for a cast Cr-Mo-V steel at high temperature condition (823 K). However, they did not discuss the mechanisms of those processes^15,36^.

In the case of an extremely low loading frequency (0.01 Hz or less), it is assumed that the fracture surfaces are exposed to air for a long period of time; therefore, oxidation might be enhanced, especially in high oxidation rate conditions as moist, high temperature and other conditions. Therefore, at an extremely low loading frequency, a higher frequency may be beneficial for decreasing the oxide-induced closure.

**Figure 7:**
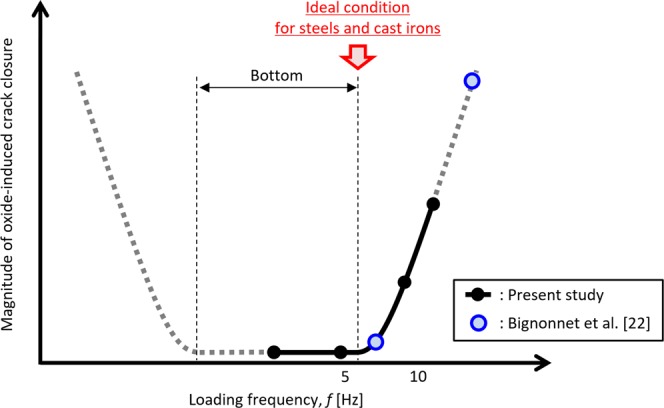
Summary of the effects of loading frequency on oxide induced crack closure.

On the other hand, Todd *et al*^30^. reported that the Δ*K*_th_ of MIL-S-24645 steel obtained at 0.2 Hz is lower than that at 10 Hz, and the two reports by Todd *et al*^30^. and Radon^36^ contradict each other. Differences in the properties of the materials used in these studies (easy to oxidize or not) or in the testing environments (moist or not) may have occurred, leading to the contradictory results. Therefore, it is thought that both findings (that low frequencies can be beneficial for avoiding the oxide-induced closure^22,27,30^ or not beneficial^15,36^) are true depending on the testing conditions. However, these past studies missed that the curve of the effect of the loading frequency on the oxide-induced closure is concave, as shown in Fig. 7.

The effect of the loading frequency on oxide-induced crack closure is summarized in Fig. 7. Most notably, the curve is concave, which means that oxide-induced crack closure is negligible got that condition. In addition, the results from the three different materials in this study suggest that almost all steels and cast irons might have an ideal condition, meaning that the ideal condition consists of an upper frequency of approximately 5 Hz where the effects of oxide-induced crack closure are minimized.

## Conclusions

In this study, the conditions necessary to minimize oxide-induced crack closure in the near threshold region were found using the Δ*K*-decreasing tests for a low alloy steel, a carbon steel, and cast iron under varying loading conditions. The results for the three different materials suggest that other iron-based materials also have the same conditions.

Furthermore, it was confirmed that the testing conditions determines the shape of the crack-front line near the threshold region and can be influenced by controllable parameters such as plasticity-induced crack closure and the residual stress of the specimen. Thus, we could realize the very small and ideal surface defects employing the pre-cracked specimen with a reproducible crack front shape in the near threshold region.

## Methods

### Materials and specimen

The materials used in the present study were a low alloy steel (JIS-SCM440), a carbon steel (JIS-S50C) and cast iron. The chemical compositions are shown in Table 1. The micro-structures of these samples are martensite, ferrite/pearlite and flake graphite with ferrite/pearlite, respectively. The Vickers hardness (2 kgf/30 sec) of the low alloy steel and the carbon steel are 437 and 195, respectively. The Brinell hardness (735 kgf / *Φ* 5 mm) of the cast iron is 219. These materials were chosen because they have different characteristics and because they are the most popular materials used for mechanical structures.

**Table 1:** Chemical compositions of the materials [wt. %].

	C	Si	Mn	P	S	Cu	Ni	Cr	Mo	V	Sb
Low alloy steel	0.38	0.18	0.64	0.014	0.002	0.12	0.07	1.04	0.15	0.01	−
Carbon steel	0.48	0.18	0.63	0.014	0.01	0.14	0.05	0.06	0.02	0.01	−
Cast iron	3.51	2.32	0.71	0.052	0.085	−	−	0.02	−	−	0.023

In order to study the effect of the thickness of specimen on the test results, we used two compact tension (CT) specimens^1^ with the width *W* = 50.8 mm, one with the thicknesses *B* = 12.7 mm and the other 6 mm. To decrease the effect of the residual stress on the test results, the specimens were machined using the wire electric discharge machining and the surfaces were finished by polishing. To avoid the pre-crack surface to be exposed to air for a long period of time, the specimens were pre-cracked just before the testing.

### Fatigue crack growth testing

The tests were carried out using the electrohydraulic servo fatigue testing machine. The stress ratio was 0.1. To exclude the frequency effect on the plastic deformation near the crack tip, the sinusoidal wave was employed.

The loading frequencies were chosen to be 20, 10, 5 and 3 Hz. The reasons why they were employed were as follows, 20 Hz: the upper limit of our testing system, 10 Hz: for comparison with the result of Tokaji *et al*.^26^, 5 Hz: for comparison with the result of Tazoe *et al*.^37^, and 3 Hz: the lower limit due to the allowance of testing time.

To minimize the effects of the plasticity-induced crack closures on the test results, the Δ*K*-decreasing conditions based on ASTM standards^1^ were employed. During the tests, the crack lengths, *a*, were measured using the compliance method with a clip gage (MTS 632.03F-30) and the *K* value was calculated as follows,

4 $$K=\frac{{P}_{load}}{B\sqrt{W}}\frac{(2+\alpha )}{{(1-\alpha )}^{3/2}}(0.886+4.64\alpha -13.32{\alpha }^{2}+14.72{\alpha }^{3}-5.6{\alpha }^{4})$$

where *P*_load_ is the fatigue load, *α* = *a*/*W*, and the normalized *K*-gradient, *C* = (1/*K*)(d*K*/d*a*), is kept larger than −0.08 mm^−1^.

The environment of the testing room was controlled by a gas heat pump system and the temperature and humidity were set to be approximately 298 K and less than 30%, respectively.

To analyse the fracture surface, the tested specimens were soaked in liquid nitrogen and broken along the loading direction. The broken specimens were soaked in isopropyl alcohol (99.7%) immediately and warmed to room temperature without dew condensation.

### Equipment and settings

The macroscopic fracture surfaces (shown in Figs. 1 and 6) are observed by optical microscope. The magnification was 20. The EDX analysis (shown in Figs. 2 and 3d–g) was carried out using a Horiba-Oxford EMAX-EX series detector. The accelerating voltage of SEM was 15 kV. The SEM images (shown in Fig. 3a–c) are also captured with the accelerating voltage of 15 kV.
